# Validity of the Perceived Physical Ability Scale for Children: An Actigraphic Study

**DOI:** 10.3390/ijerph182211900

**Published:** 2021-11-12

**Authors:** Lorenzo Tonetti, Alicia Carissimi, Marco Fabbri, Marco Filardi, Sara Giovagnoli, Monica Martoni, Vincenzo Natale

**Affiliations:** 1 Department of Psychology “Renzo Canestrari”, University of Bologna, 40127 Bologna, Italy; sara.giovagnoli@unibo.it (S.G.); vincenzo.natale@unibo.it (V.N.); 2 Laboratório de Cronobiologia e Sono do Hospital de Clínicas de Porto Alegre (HCPA), Universidade Federal do Rio Grande do Sul (UFRGS), Porto Alegre CEP 90035-003, RS, Brazil; alicia.ufrgs@gmail.com; 3 Faculdade Dom Bosco de Porto Alegre, Porto Alegre CEP 90520-280, RS, Brazil; 4 Department of Psychology, University of Campania Luigi Vanvitelli, 81100 Caserta, Italy; marco.FABBRI@unicampania.it; 5 Department of Basic Medicine, Neuroscience and Sense Organs, University of Bari “Aldo Moro”, 70121 Bari, Italy; marco.filardi@uniba.it; 6 Center for Neurodegenerative Diseases and the Aging Brain, Department of Clinical Research in Neurology, University of Bari “Aldo Moro” at “Pia Fondazione Cardinale G. Panico”, 73039 Lecce, Italy; 7 Department of Experimental, Diagnostic and Specialty Medicine, University of Bologna, 40138 Bologna, Italy; monica.martoni@unibo.it

**Keywords:** physical self-efficacy, actigraphy, children, perceived physical ability scale for children, 24 h motor activity pattern

## Abstract

This study aimed to provide evidence of the validity of the Perceived Physical Ability Scale for Children against an external-objective criterion of the 24 h motor activity pattern assessed through actigraphy. A total of 107 children (60 females; mean age 10.25 ± 0.48) were originally enrolled. Children wore the actigraph model Actiwatch AW64 (Cambridge Neurotechnology Ltd., Fenstanton, UK) for seven days, 24 h per day, around the non-dominant wrist. At the beginning of the actigraphic recording, participants filled in the Perceived Physical Ability Scale for Children. Functional Linear Modeling was used to examine variation in the 24 h motor activity pattern according to the total score in the Perceived Physical Ability Scale for Children. Higher physical self-efficacy was significantly related to greater levels of motor activity in the afternoon. Overall, this pattern of results supports the validity of the Perceived Physical Ability Scale for Children against the external-objective criterion of the 24 h motor pattern. The Perceived Physical Ability Scale for Children could represent a promising endpoint for studies assessing the effectiveness of physical activity promotion interventions.

## 1. Introduction

Self-efficacy [1,2] refers to the beliefs and expectations regarding one’s ability to organize and successfully conclude a requested action in future contexts and tasks, i.e., beliefs and expectations in terms of one’s own performance. A specific form of self-efficacy is within the physical self-efficacy domain [3], i.e., beliefs regarding one’s own ability to effectively undertake a physical activity with some intensity, duration, and frequency. It has been previously reported that physical self-efficacy plays a significant predictive role of actual physical activity engagement [4], both in healthy children [5,6] and in children suffering from congenital heart disease [5,7]. Moreover, it has been clearly pointed out that programs of childhood obesity prevention should strengthen the psychosocial correlates of physical activity [8]. Among these correlates, physical self-efficacy is commonly considered one of the most relevant determinants of physical activity engagement [9].

Given the important predictive role of actual physical activity played by physical self-efficacy, the assessment of physical self-efficacy is of utmost importance in order to develop interventions aimed at promoting this psychological construct in those who present critically low levels. Among the questionnaires available to assess physical self-efficacy in children, the Physical Self-Efficacy Scale for Children, originally proposed in Italian by Colella and colleagues [10], is considered for the purpose of this study. They administered the Physical Self-Efficacy Scale for Children to 1914 children aged between 8 and 10 years, showing that this tool has adequate psychometric properties in terms of internal validity and reliability. This scale was also translated into Arabic by Abd-El-Fattah [11], who reported a positive correlation between the total score of the Physical Self-Efficacy Scale for Children and in-class physical activity, recorded through activity monitor devices during physical education classes (higher physical self-efficacy was associated with higher motor activity levels).

The aim of the current study is to provide further evidence of the validity of the Physical Self-Efficacy Scale for Children by using objective motor activity, recorded through actigraphy, as an external criterion. More in detail, variation in the 24 h motor activity pattern, i.e., the minute-by-minute motor activity over 24 h, will be examined against the total score of the Physical Self-Efficacy Scale for Children. If the Physical Self-Efficacy Scale for Children proves to have a good validity, we could expect an association between higher physical self-efficacy and higher motor activity recorded through actigraphy during the daytime.

## 2. Materials and Methods

### 2.1. Participants

A total of 107 Italian children, 60 females and 47 males, took part in a larger project [12,13,14] on the potential roles played by rest/activity cycle, motor activity pattern, and timing of food intake in the variation of body mass index in childhood, entitled “Body mass index in childhood: activity/rest cycle, motor pattern and food timing”. Children were attending primary schools located in the Emilia-Romagna region (Italy) over the school year 2013/2014 from Monday to Friday (8:30–16:30).

### 2.2. Perceived Physical Ability Scale for Children

The Perceived Physical Ability Scale for Children (PPASC [10]), aimed at measuring physical self-efficacy, is an adaptation of the Perceived Physical Ability subscale of the Physical Self-Efficacy scale by Ryckman et al. [15]. The original subscale was composed of 10 items (Italian translation for children and young people by Bortoli and Robazza [16]), while the version by Colella and colleagues [10] identified the 6 most representative items, measured on a four-level Likert Scale, regarding the perception of coordinative abilities, speed, and personal strength. For each item, the replies range from the lowest (1) to the highest (4) perception. The total score of the PPASC, ranging from 6 to 24, is obtained by adding up the scores of each item, with higher scores indicating higher physical self-efficacy. An example of one item taken from the PPASC concerns running, with the following replies: “I run very slowly” (1), “I run slowly” (2), “I run fast” (3), “I run very fast” (4).

### 2.3. Actigraphy

The actigraph Actiwatch AW64 (Cambridge Neurotechnology Ltd., Fenstanton, UK) was used in order to measure motor activity. The actigraph is equipped with a piezoelectric accelerometer with a sampling frequency of 32 Hz and sensitivity of ≥0.05. The actigraphs were initialized through version 5.32 of the Actiwatch Activity and Sleep Analysis software (Cambridge Neurotechnology Ltd., Fenstanton, UK) to collect data in 1 min epochs, with the wake sensitivity set to the low threshold [17,18].

### 2.4. 24 h Motor Activity Pattern

Version 5.32 of the Actiwatch Activity and Sleep Analysis software (Cambridge Neurotechnology Ltd., Fenstanton, UK) was used to extract the raw motor activity counts, minute-by-minute, over the 24 h period of each school day. Then, for each participant, the mean of the school days was computed, allowing us to describe the raw mean 24 h motor activity pattern.

### 2.5. Procedure

Participants were requested to wear the actigraph around the non-dominant wrist for seven days, 24 h per day, during a regular school period. At the beginning of the recording week, children had to fill in the PPASC.

Parents provided written informed consent to allow their children to participate in the study, which was approved by the Bioethics Committee of the University of Bologna (report number 2.10 of 11.9.2013).

### 2.6. Statistical Analysis

Functional Linear Modeling (FLM [19]), a statistical framework specifically developed for the analysis of data acquired through actigraphy, was used to examine the variation in the 24 h motor activity pattern according to the PPASC total score. The raw 24 h motor activity pattern is replaced with a function by applying the Fourier expansion model and is then analyzed through a non-parametric permutation F-test to ascertain a significant association between motor activity levels and PPASC total score.

## 3. Results

The mean age of the complete sample was equal to 10.25 (SD = 0.48; range = 9–11 years old). Females (M = 10.18; SD = 0.50) did not significantly differ in age from males (M = 10.34; SD = 0.44) (t_105_ = −1.76; *p* = ns).

In the whole sample, the mean total score of the PPASC was 18.43 (SD = 2.44). Females reported significantly lower total scores (M = 17.82; SD = 2.15) compared to males (M = 19.21; SD = 2.58) (t_105_ = −3.05; *p* < 0.005).

**Figure 1:**
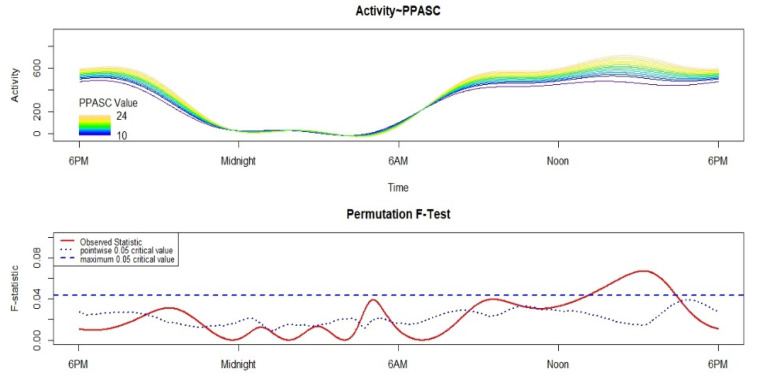
Results of the Functional Linear Modeling applied to the analysis of the variation in the 24 h motor activity pattern according to the total score of the Perceived Physical Ability Scale for Children (PPASC). The upper panel shows the circadian activity rhythm plotted according to PPASC, with different colors depicting the circadian activity rhythm of participants with different PPASC total scores. Blue indicates the lowest total score of the PPASC, while dark yellow refers to the highest. The lower panel shows the results of the non-parametric permutation F-test. Significant differences can be detected when the solid red line is above the dotted blue line (i.e., the point-wise test of significance) or the dashed blue line (i.e., the global test of significance, more conservative).

The results of the FLM analysis are reported in Figure 1. Considering the most conservative test of significance, higher physical self-efficacy is associated with higher motor activity in the afternoon between 13.00 and 16.30.

Given the significant gender difference in physical self-efficacy, the variation in the 24-motor activity pattern according to the PPASC total score was examined separately by gender. No significant association between physical self-efficacy and motor activity over the 24 h period was detected either in males or in females (data not shown).

## 4. Discussion

The aim of the current study was to provide further evidence of validity of the PPASC using motor activity, recorded through actigraphy, as an external criterion. To this end, the variation in 24 h motor activity pattern according to the PPASC total score was examined through FLM in an Italian sample of children with quite a similar age range (i.e., 9–11 years old) compared to that of the Italian sample of children being examined in the original validation study of the PPASC (i.e., 8–10 years old) by Colella and colleagues [10].

The main result of the current study, overall in line with our expectations, is the association between higher physical self-efficacy and increased levels of motor activity in a specific time window over a 24 h period, i.e., in the afternoon when arousal is higher, although other variations of motor activity according to the PPASC total score were observed over the 24 h (for example, during the morning window) without reaching the highest level of significance. Indeed, it is well known in the literature that in the second half of the day an increase in arousal can be observed [20]. Another possible explanation of this result is that the relationship between physical self-efficacy and motor activity manifests only when participants can start to behave more freely, due to the decreasing scholastic load. Overall, this pattern of results supports the validity of the PPASC against an objective external criterion of 24 h motor activity pattern and is somewhat in line with previous evidence available in the literature, although the study by Abd-El-Fattah [11] focused only on motor behavior assessed during physical education classes. Previous studies assessed motor behavior through field-based tests instead of actigraphy. For example, Colella and colleagues [21] assessed physical self-efficacy through the PPASC and motor performance through field-based tests in non-overweight and overweight children. They reported lower physical self-efficacy and lower motor performance in one of the tasks in overweight children compared to non-overweight children. A similar pattern of results has been observed by a recent study [22] showing poorer motor performance and lower physical self-efficacy in overweight and obese children than normal-weight children. Although the current study used actigraphy to measure motor activity over the 24 h while previous works used actigraphy (i.e., [11]) or in-field based tests (i.e., [21,22]) to assess motor behavior in a limited timeframe, overall, the resulting pattern of results seems to confirm that higher physical self-efficacy is significantly related to higher motor activity.

Since some evidence of the external validity of the PPASC is available, the present study could have some applied implications within the field of childhood obesity prevention. Namely, since physical self-efficacy can predict engagement in physical activity that reduces the risk of developing obesity and metabolism disorders, this scale could be usefully administered for early detection of low physical self-efficacy in children. These high-risk children could then take part in programs that are specifically developed to strengthen their physical self-efficacy [23]. In addition to the role in the early detection of high-risk children, this scale could also be helpfully used in assessing the effectiveness of interventions aimed at promoting physical activity in children [24]. We are fully aware that other important dimensions are involved in the compliance to a complex behavioral recommendation as the practice of physical activity; therefore, the role played by physical self-efficacy, highlighted in the current study, is just a piece of a complex puzzle. To this regard, the concept of physical literacy (“a learning process which allows, to all kids, to acquire a linguistic motor repertoire according to individual rhythms of maturation and growth and in relationship to the opportunities offered by the external environment (family, school, society) starting from fundamental motor skills, in all ages groups”; [25]; page 2) can be quoted. It is interesting that a multimodal intervention, including, among different action plans, physical literacy, showed as the main outcomes an improvement in motor performance in primary school-aged children [25]. Underlying the complexity of the promotion of healthy lifestyles in children, another multicomponent program [8], based on the combination of interventions at physical, psychosocial, and behavioral levels, has proven to be effective in reducing body mass index and increasing both the actual and perceived physical abilities in overweight and obese children.

The gender difference reported in the current study, with males presenting higher physical self-efficacy compared to females, is in line with the literature [10,13,25]. It is possible that gender stereotypes in physical activity [26] could lead to a greater involvement in physical activity by males, which could ultimately promote the development of more confidence in their own ability.

Among the limitations, the reduced age range and the relatively small sample size can be quoted. In order to overcome these limitations, future studies could try to examine samples that are larger in size and with a larger age range within childhood populations. Despite these limitations, the present study increases the available knowledge concerning the validity of a subjective measure, such as the PPASC, explored through the objective external criterion of motor activity.

## 5. Conclusions

An association between higher physical self-efficacy and higher levels of motor activity was observed in a limited time window over 24 h, leading to the conclusion that the PPASC presents good validity against the objective external criterion of 24 h motor activity pattern assessed through actigraphy.

## Data Availability

The data are not publicly available and cannot be shared due to ethical issues.
